# Outcomes and associated factors of cervical human papillomavirus infection among 608 women in Shenzhen, China, 2018–2023

**DOI:** 10.3389/fpubh.2024.1523839

**Published:** 2025-01-14

**Authors:** Zhenghan Lv, Xuesen He, Zhiju Li, Yue Yuan, Xinyi Zhou, Changqing Tu, Yinqi Yang, Yanshan Huang, Lili Yin, Huihui Chen, Yanling Tao

**Affiliations:** ^1^Clinical Medical College of Shenzhen, Guangzhou University of Chinese Medicine, Shenzhen, China; ^2^Department of Preventive and Health Care, Longgang District Central Hospital of Shenzhen, Shenzhen, China; ^3^Longgang District Central Hospital of Shenzhen, Shenzhen, China

**Keywords:** human papillomavirus, outcome, prognosis, clearance rate, cervical lesions, negative conversion

## Abstract

**Objective:**

This study aimed to uncover the patterns of Human papillomavirus (HPV) infection outcomes in women and assess the risk factors that may affect these outcomes.

**Methods:**

A retrospective study was conducted on 608 women who tested positive for HPV-DNA during their initial visit to the outpatient department of Shenzhen Longgang Central Hospital from 2018 to 2023 and who had subsequent HPV-DNA testing as part of their post-visit monitoring. The monitoring intervals were every 6 months. The rank sum test was used to analyze ranked data. The Kaplan–Meier method was used to analyze the turning negative time. Univariate analysis was performed using the log-rank test, and multivariate analysis was performed using the Cox model to analyze independent risk factors.

**Results:**

The results showed that the median age was 40.00 years (interquartile 33.00–47.00 years), the total conversion rate of the HPV-negative patients was 38.98%, and the median conversion time of the HPV-negative patients was 8.95 months (interquartile 4.20–16.175 months). Age, infection status and type of health insurance were significantly correlated with HPV outcome (*p* < 0.05).

**Conclusion:**

Among women infected with HPV, the overall rate of negative HPV infection was 38.93%, and the duration of negative conversion was 8.95 months. The study revealed that age, HPV infection status, and type of medical insurance are independent predictors of the persistence of negative HPV test outcomes.

## 1 Introduction

Human papillomavirus (HPV) is the most prevalent sexually transmitted disease, affecting more than 70% of sexually active women throughout their lifetime (1–3). A meta-analysis (4) reported a total HPV infection rate of 14.3% among women with a normal cervix in China, with a prevalence rate of 12.1% in South China. Further studies (5, 6) revealed HPV infection rates of 21.66 and 19.33% in Guangzhou and Shenzhen, respectively. Genital warts are associated primarily with low-risk human papillomavirus (LR-HPV) types 6 and 11, whereas high-risk human papillomavirus (HR-HPV) types 16, 18, 31, 33, 45, 52, and 58 are linked to cervical cancer (7–10). According to GLOBOCAN 2022 statistics, about 660,000 individuals were diagnosed with cervical cancer, and about 350,000 succumbed to the disease worldwide in 2022, with the majority—more than 85%—of these incidents occurring in developing nations (11, 12). In China, according to the National Cancer Center’s 2022 data, 150,700 incident cases of cervical cancer and 55,700 fatalities have been recorded, comprising 85,500 new cases and 30,200 deaths in urban areas and 65,200 new cases and 25,500 deaths in rural areas (13). A predictive study by Duan et al. (14) forecasts an increase in the number of HPV-associated cancer cases from 117,929 to 214,077 between 2016 and 2030, with age-standardized rates rising from 6.07 to 9.35 per 100,000 people. Cervical cancer is projected to account for 87.7% of all HPV-associated cancers. The burden of HPV-associated cancers in China is anticipated to continue rising over the next decade, imposing significant mental stress and disease on women.

Epidemiological research has shown that the majority of HPV infections are cleared spontaneously within 2 years due to autoimmune responses, although about 10–20% of women experience persistent infection (1, 4, 15). The course of HPV infection is affected by a combination of personal and environmental factors, which contributes to the substantial differences in clearance rates observed across regions (10, 16, 17). Vaccination with the HPV vaccine is an effective preventive measure against HPV infection (18–20). However, for individuals who are already infected, the clinical efficacy of anti-HPV drugs remains unclear, and further verification of the effectiveness of therapeutic HPV vaccines is needed (21–23). Additionally, the effect of physical therapy on HPV clearance varies significantly across individuals (24). Grasping of HPV infection outcomes within particular demographic groups is essential, enabling healthcare providers to effectively track and treat individuals with HPV. This knowledge also holds significant importance for the dynamic management of cervical lesions associated with HPV and for cervical cancer, underscoring the necessity for region-specific research and tailored intervention strategies.

While numerous studies have outlined the mechanisms of HPV infection, the processes of HPV clearance and the factors that influence this remain unclear. Particularly, debates persist regarding the outcomes of HPV coinfection, with significant variation observed across different countries, regions, economic statuses, and ethnic groups. To address these gaps, we propose a study on HPV outcomes in one of China’s largest and youngest cities, aiming to examine the patterns of HPV outcomes in urban Chinese women.

This study used Shenzhen Longgang Central Hospital as the center, which was selected from 2018 to 2023 outpatient women with HPV infection for investigation and follow-up. To explore the outcome of female genital HPV infection, including the HPV infection negativity rate and negative time, the general demographic information, test results, and pathological results of patients were collected through the medical records system, and the related risk factors affecting the development of infection negativity were analyzed. These findings will contribute to a better understanding of the outcomes and related factors of HPV infection in women in Shenzhen, China. Moreover, these findings can provide a basis for the clinical diagnosis and treatment, disease management, and prognosis follow-up of HPV-infected women.

## 2 Objects and methods

### 2.1 Object

The selected patients were admitted to Shenzhen Longgang Central Hospital from 2018 to 2023, according to the expert consensus on HPV infection and HPV-associated diseases (25). For women diagnosed with HPV infection, patient information was obtained through an electronic medical record system. The inclusion criteria were as follows: (1) were women aged ≥18 years; (2) were married or unmarried patients with a sexual life history; (3) were in line with the Western diagnosis of human papillomavirus infection; (4) had complete clinical data; and (5) had two or more HPV-DNA tests. The exclusion criteria were as follows: (1) pregnant or lactating; (2) mental disorders or consciousness disorders; and (3) serious organ damage and related functional damage to the heart, liver or kidney. The study sample comprised 608 female participants who satisfied the inclusion criteria.

This study was conducted in accordance with relevant guidelines, regulations, and principles outlined in the Declaration of Helsinki. The Ethics Committee of Shenzhen Longgang Central Hospital reviewed and approved the study protocol (Approval Number: 2024ECPJ004). Written informed consent was obtained from all participants prior to their involvement in the study, ensuring compliance with ethical standards for human subject research.

To ensure participants’ privacy and data security, this study strictly adheres to data protection regulations. All collected data is de-identified, and participants’ personal and health information is kept confidential throughout the analysis process. Research data is restricted to authorized personnel and stored in an encrypted database. All analyses are conducted on anonymized data to prevent the disclosure of any personal identities. Furthermore, all research data will be securely destroyed upon completion in accordance with Shenzhen Longgang Central Hospital’s data management policies. Any publicly available data will be fully de-identified and limited to aggregated results necessary for dissemination. Under no circumstances will the research data be shared with unrelated third parties, ensuring the full protection of participants’ privacy rights.

### 2.2 Methods

Data collection: Age, ethnicity, occupation, ancestral place, home address, medical insurance type, marital status, birth history, abortion history, menopausal status, use of intrauterine devices, HPV infection status, cervical lesion degree, treatment method, and HPV outcome data were collected from the hospital medical record system. According to the correlation with the degree of cervical intraepithelial neoplasia (CIN), the 28 detected HPV types were classified as high-risk types (16, 18, 31, 33, 35, 39, 45, 51, 52, 53, 56, 58, 59, 66, 68, 73, 82) or low-risk types (6, 11, 23,40,42, 43, 44,54,61,81, 83). According to the correlation with CIN, the patients were divided into high-risk HPV (HR-HPV) infection (regardless of how many types of HPV they were infected, positive results included only HR-HPV), low-risk HPV (LR-HPV) infection (regardless of how many types of HPV they were infected, positive results included only LR-HPV), and mixed infection (regardless of how many types of HPV they were infected, positive results included both HR-HPV and LR-HPV). The participants in the study were categorized into 5 distinct groups based on the severity of their cervical lesions: normal smear, CIN I, CIN II, CIN III and cervical cancer. The number of infected HPV strains can be divided into single infections and multiple infections, which can be divided into two infections, three infections or more. Follow-up: Follow-up was conducted via telephone, and patients returned to the hospital regularly for review. The follow-up time ranged from the first visit date to June 2024, and the follow-up interval was once every 6 months. The follow-up data concerned the outcomes of the HPV-infected women, and the outcomes were divided into negative and persistent infections. If the HPV-DNA test is negative at the time of review, the HPV infection of the woman is considered negative; persistent HPV infection is diagnosed if a woman’s test results remain positive, indicating the need for further evaluation and management.

### 2.3 HPV-DNA testing

For women with normal menstruation, 10–18 days after menstruation is the best time for examination; vaginal irrigation should be avoided within 3 days before the examination; the use of contraceptive ointment and other vaginal drugs should be avoided; no sexual activity should occur within 24 h of the test; and the application of acetic acid or iodine solution should be contraindicated prior to the examination.

A special cervical exfoliation cell collector was used for sampling. The gynecologist first exposed the cervix with a voyeur or vaginal opener and then wiped the excessive secretions from the cervix with a cotton swab. The cervical brush was removed and placed in the cervical opening. The tip of the cervical brush was rotated 4–5 times in one direction to obtain sufficient epithelial cell samples. The tip of the cervical brush was then placed into the eluting tube, the handle was broken along the crease of the handle, the cover of the eluting tube was tightened, the sample was marked, and the eluting tube was kept upright. Once collected, the samples were submitted for examination as soon as possible: the samples were stored at room temperature for no more than 12 h, at 4°C for no more than 7 days, and at −20°C for no more than 3 months. Repeated freezing and thawing cycles were avoided. The foam box and ice bag were sealed, and the transit time did not exceed 48 h.

A human papillomavirus (HPV) genotyping test kit (Yaneng Biotechnology Co., Shenzhen, China) was utilized to identify 28 HPV types, comprising 17 HR-HPV types (HPV 16, 18, 31, 33, 35, 39, 45,51, 52, 53, 56, 58, 59, 66, 68, 73, 82) and 11 LR-HPV types (HPV 6, 11, 23, 40, 42, 43, 44, 54, 61, 81, 83). Genotyping of HPV was conducted through PCR-based amplification followed by DNA reverse line blot hybridization. Specifically, designed primers targeted 23 HPV genotypes, amplifying specific fragments. The amplification products were then hybridized with 17 high-risk and 6 low-risk probes fixed on a membrane. The presence of hybridization signals indicated HPV genotype infection. The testing process consisted of HPV-DNA extraction, PCR amplification, hybridization, membrane washing, color rendering, and result analysis. Both positive and negative controls, provided in the kit, were used during testing according to the manufacturer’s instructions.

### 2.4 Statistical methods

Data processing and statistical analysis were conducted using SPSS version 27.0. Normally distributed data are presented as ^−^X ± S values, with group comparisons performed via independent samples *t*-tests or chi-square (X^2^) tests. The non-normally distributed measurement data are represented by *M (P25–P75)*, and the rank-sum test was used for comparisons between groups. Count data are represented as *N* (%). Kaplan–Meier survival analysis, the log-rank test and the Cox proportional risk regression model were used for survival analysis and influencing factor analysis. In this study, *p* < 0.05 was considered statistically significant.

The HPV-negative conversion rate was calculated as the proportion of women who cleared their HPV infection during follow-up. The calculation was based on the following: denominator: the total count of women who had a positive HPV-DNA result during their initial visit (2018–2023) and subsequently underwent re-evaluation. Numerator: the number of women with first-time HPV-DNA detection (2018–2023) and who subsequently tested negative for HPV-DNA during follow-up as the numerator.

## 3 Results

### 3.1 General information

The study included a sample of 608 HPV-infected women who visited the clinic between 2018 and 2023. The median age at initial diagnosis was 40.00 years (quartile 33.00–47.00 years) and ranged from 18 to 73 years. Among the 608 women, 237 patients (38.98%) were negative, and 371 (61.02%) were still infected at the end of the follow-up period. The median duration of negative HPV results was 8.95 months (quartile range 4.20–16.175 months), with a range of 1.1–58.1 months. Among the 608 enrolled women, 169 (27.8%) had normal smears at initial diagnosis; 102 (16.8%) were diagnosed with CINI. In total, 37 patients (6.1%) had CIN II, 292 patients (48%) had CIN III, and 8 patients (1.3%) had cervical cancer. The rank-sum test revealed statistically significant differences in age, ethnicity, occupation and treatment mode among groups with different cervical lesions (*p* < 0.05), as shown in Table 1. No substantial variations were observed in the infection types, incidence of multiple infections, or levels of infection risk across groups characterized by distinct cervical lesions (*p* > 0.05), as shown in Table 2.

**Table 1:** Baseline demographic, behavioral, and health characteristics of 608 HPV-infected women at Shenzhen Longgang Central Hospital from 2018 to 2023, divided by the degree of cervical lesions.

Variable		Grouping				*H*	*p* ^b^
		Normal smear and CIN I^a^	CIN II^a^	CIN III^a^	Cervical cancer		
Age	<30	45	9	29	0	7.849	<0.05
	30–60	219	28	258	7		
	>60	7	0	5	1		
Gestational number	1–2	106	9	132	2	3.291	0.349
	>3	142	24	154	6		
	0	23	4	6	0		
Yield	1–2	186	26	221	5	6.211	0.102
	>3	51	6	57	3		
	0	34	5	14	0		
Ethnicity	Han nationality	267	35	272	7	10.893	<0.05
	Ethnic minorities	4	2	20	1		
Matrimony	Married	237	32	258	7	0.174	0.982
	Unmarried	34	5	34	1		
Occupation	Enterprise business service personnel	29	2	8	0	60.873	<0.01
	Workman	36	1	62	0		
	Farmer	40	13	100	2		
	Otherness	122	11	46	2		
	People in state organs, party organizations, public institutions	22	4	19	1		
	Out of work	22	6	57	3		
Ancestral place	Out of province	175	18	166	5	5.560	0.135
	Province	96	19	126	3		
Current address	City	175	21	192	2	6.548	0.088
	Rural area	96	16	100	6		
Medical insurance Category^c^	Shenzhen medical insurance	189	23	215	6	1.505	0.681
	Self-pay	66	11	44	1		
	Offsite^d^	16	3	33	1		
History of abortion	No	124	12	125	2	3.560	0.313
	Yes	147	25	167	6		
Menopause	No	209	31	243	5	5.173	0.160
	Yes	62	6	49	3		
Intrauterine devices	No	200	31	210	6	2.403	0.493
	Yes	71	6	82	2		
State of transition	1	117	15	104	1	5.779	0.123
	0	154	22	188	7		
Treatment mode	No	72	3	0	0	364.838	<0.01
	Pharmacotherapy	168	15	24	0		
	Surgical treatment	31	19	268	8		

a CIN I denotes mild, CIN II indicates moderate, and CIN III signifies severe cervical cell abnormalities, with the associated cancer risk escalating in accordance with the severity of the grade.

b *p values of infection differences between different cervical lesions.*

^cShenzhen medical insurance is divided into three levels, which are applicable to different insured persons: I. First-level medical insurance is mainly for deep-household employees, who are jointly paid by the unit and individual. The outpatient and hospitalization reimbursement ratio is high. The insured can be in the city at any designated hospital for medical treatment without binding to a social health center. II. Second-tier health insurance: applicable to non-deep-household employees and other voluntary alternatives. Outpatient reimbursement is low, requiring binding to a social health center. The hospitalization reimbursement ratio is low, and out-of-city hospitalization requires referral procedures. III. Three-tier medical insurance: resident medical insurance, applicable to unemployed residents, students, etc. The payment is the lowest, and the reimbursement ratio is similar to that of the second grade. You need to bind to the social health center for outpatient reimbursement.^

d Remote medical insurance means that the insured can still enjoy medical insurance benefits when they seek medical treatment in places other than the insured place. It solves the problem of medical security for people from different regions, including direct settlement of medical treatment in other places and medical insurance services in long-term residence. The main forms of long-distance medical insurance are as follows: I. Long-distance medical settlement: the insured can directly settle the medical expenses through the designated hospitals in other places without the need to pay in full. I. Off-site filing: The insured shall put on record in the medical insurance center in advance to enjoy direct settlement in other places. II. Medical insurance for long-term living in other places: it is suitable for long-term residents or retirees who can enjoy medical insurance services in their place of residence after filing.

**Table 2:** HPV infection of 608 women infected with HPV in Shenzhen Longgang Central Hospital from 2018–2023, divided by the degree of cervical lesions.

Variable		Grouping				*H*	*p*
		Normal smear and CIN I	CIN II	CIN III	Cervical cancer		
Type of infection	Single infection	204	24	198	7	5.588	0.133
	Multiple infection	67	13	94	1		
Number of multiple infections	Single infection	196	24	197	7	3.535	0.316
	Double infection	59	11	67	1		
	Triple or above infection	16	2	28	0		
Infection type and risk level	HR-HPV infection	225	31	266	7	7.635	0.054
	LR-HPV infection	21	0	5	1		
	HR and LR HPV mixed infection	25	6	21	0		

**Figure 1:**
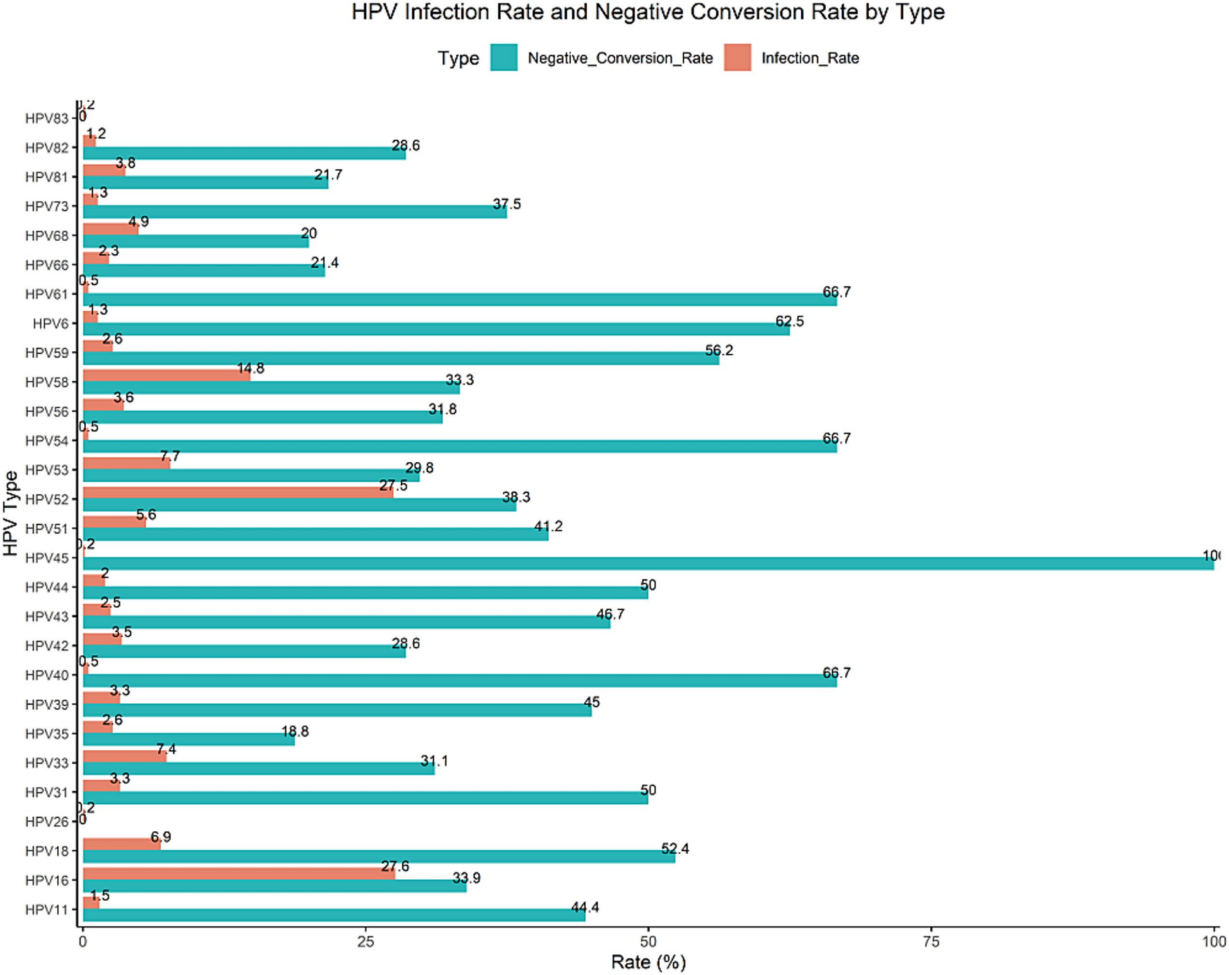
HPV infection rate and negative conversion rate by type.

**Figure 2:**
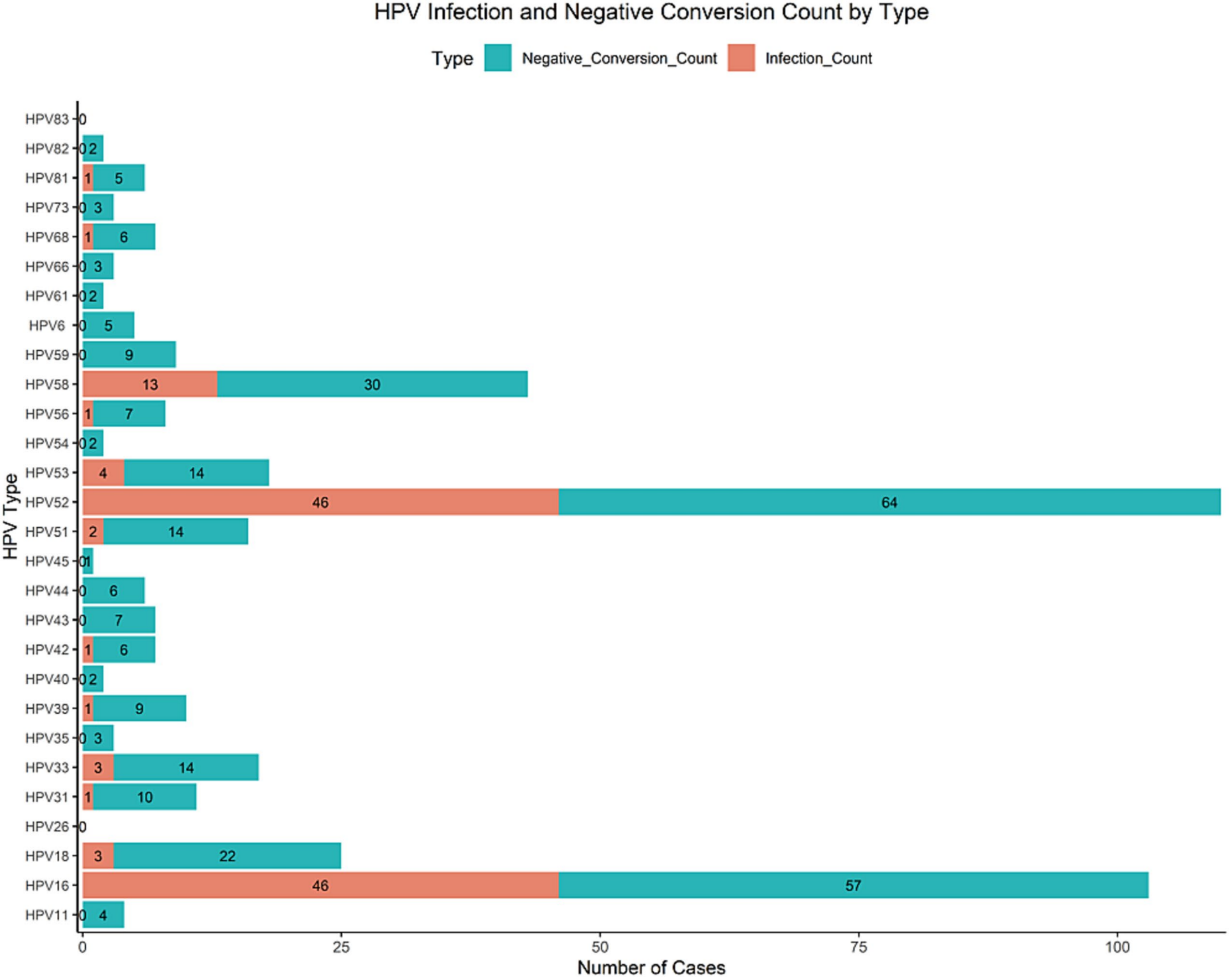
HPV infection count and negative conversion count by type.

In our study, the infection rates of HPV16 and HPV52 were the highest, at 27.632 and 27.467%, respectively, making them the most common types. HPV58 had an infection rate of 14.803%, which, while relatively high, was lower than that of HPV16 and HPV52. The infection rates of other HPV types gradually decreased, with HPV26 and HPV83 having the lowest rates at 0.164%. Overall, HPV16 and HPV52 were the predominant infection types. In terms of clearance rates, HPV6, HPV40, and HPV54 showed relatively high negative conversion rates of 62.5, 66.7, and 66.7%, respectively, indicating favorable clearance outcomes. In contrast, HPV35 had the lowest negative conversion rate at only 18.75%, suggesting a lower likelihood of clearance for this type. On the whole, the negative conversion rates of most HPV types ranged from 20 to 50%, indicating considerable variability in clearance probabilities, as shown in Figures 1, 2.

### 3.2 Prognostic analysis

#### 3.2.1 Univariate survival analysis

Univariate analysis of 608 patients with HPV infection revealed that with increasing age, the rate of HPV infection tended to decrease, and the <35 years age group had the highest rate of negative results, 46.60%. In the >50 years age group, the negative conversion rate was the lowest (28.41%). The rate of negative conversion in Han patients (39.59%) was higher than that in minority patients (25.93%). The negative conversion rate of premenopausal patients (41.39%) was significantly greater than that of postmenopausal patients (29.17%). The infection status significantly influenced the negative conversion rate, with patients exhibiting a single infection showing a notably higher rate (43.19%) than those with multiple infections (28.57%). There were differences in the HPV conversion rate and conversion duration among patients with different medical insurance types. Patients with medical insurance in Shenzhen had the highest conversion rate (42.03%). Patients with remote medical insurance had the lowest rate of negative conversion, at only 13.21%.

A correlation was observed between the conversion rate of HPV infection negativity and the severity of cervical lesions, with the conversion rate decreasing as lesion severity increased. Analysis revealed that women with normal smears had a 44.38% HPV infection conversion rate, whereas cervical cancer patients had the lowest conversion rate of 12.50%. A comparison of treatment methods revealed that patients receiving drug therapy achieved the highest negative conversion rate (50.24%), whereas those receiving surgery had the lowest rate (33.44%).

There are also differences in HPV conversion rates among women in different occupations. Among them, enterprises and commercial and service workers had the highest negative conversion rates (43.59%). Unemployed patients had the lowest rate of negative conversion (28.41%).

**Figure 3:**
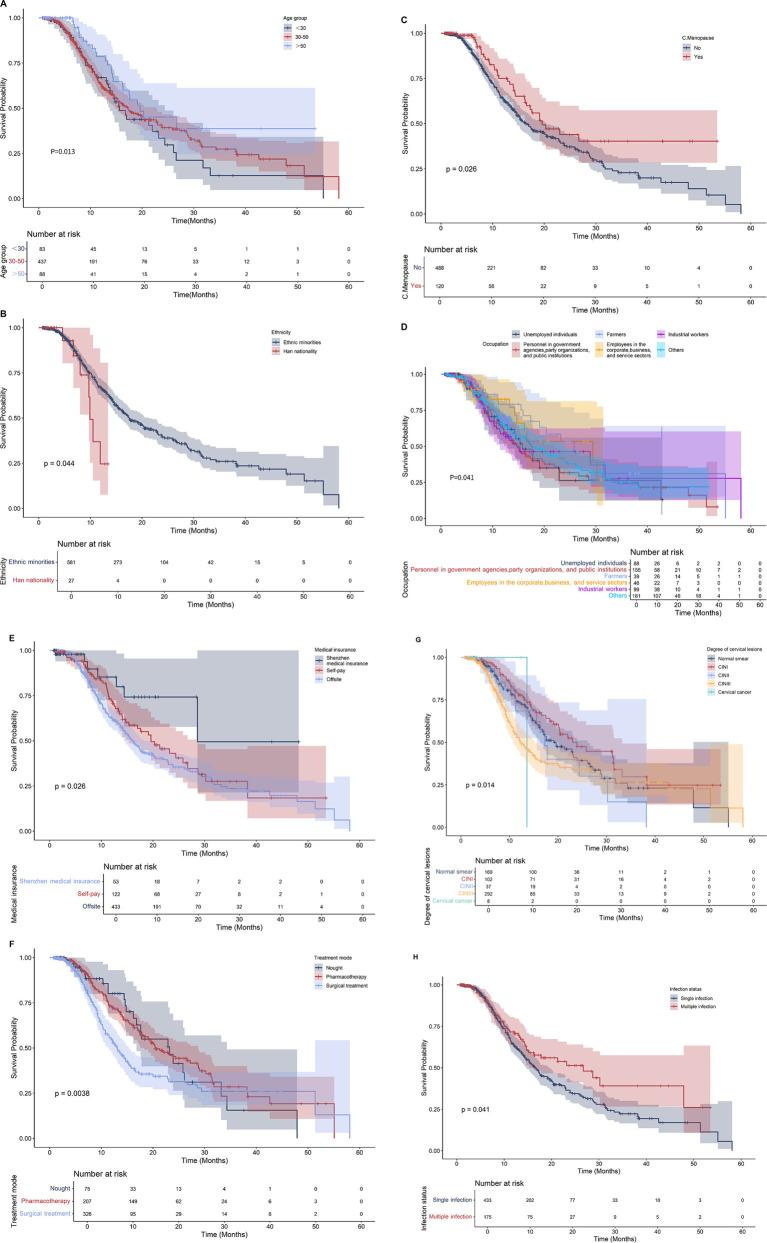
Survival curve of 608 HPV infected women. **(A)** Age; **(B)** Ethnicity; **(C)** Menopause; **(D)** Occupation; **(E)** Medical insurance; **(F)** Treatment methods; **(G)** Degree of cervical lesions; **(H)** Status of infection.

The log-rank test revealed that eight of the 17 collected variables, specifically age, ethnicity, menopausal status, infection status, medical insurance type, lesion degree, treatment mode, and occupation, demonstrated statistically significant associations with the negative conversion rate of infected patients (*p* < 0.05). The details are presented in Table 3 and illustrated in Figure 3.

**Table 3:** Single-factor analysis outcomes in 608 women with HPV infection.

Influencing factors	Total number of cases	Negative/case	Negative conversion rate (*n* %)	Median survival	X^2^	*p*
Age					6.21	<0.05
<35	206	96	46.60	14.60		
35–50	314	116	36.94	19.10		
>50	88	25	28.41	19.60		
Ethnicity					4.051	<0.05
Han nationality	581	230	39.59	17.5		
Ethnic minorities	27	7	25.93	9.9		
Matrimony					0.300	0.584
Married	534	211	39.51	17.4		
Unmarried	74	26	35.14	15.6		
Gestational number	608	237	38.98	17.3	0.16	0.21
Yield	608	237	38.98	15.55	0.43	0.356
Ancestral place					2.821	0.093
Fluidness	364	137	37.64	19.6		
Permanent residence	244	100	40.98	15.6		
Current address					0.087	0.768
City	390	157	40.26	17.8		
Rural area	218	80	36.70	15.6		
History of abortion					0.177	0.674
No	263	109	41.44	16.3		
Yes	345	128	37.10	19.1		
Menopause					4.929	<0.05
No	488	202	41.39	16.5		
Yes	120	35	29.17	19.6		
Intrauterine devices					3.544	0.060
No	447	190	42.51	16.3		
Yes	161	47	29.19	23.9		
Infection status					4.156	<0.05
Single infection	433	187	43.19	16.6		
Multiple infection	175	50	28.57	26.7		
Medical insurance Category					6.227	<0.05
Shenzhen medical insurance	433	182	42.03	16.20		
Self-pay	122	48	39.34	19.60		
Offsite	53	7	13.21	28.60		
Degree of lesion					10.87	<0.01
Normal smear	169	75	44.38	19.60		
CINI	102	42	41.18	23.90		
CINII	37	15	40.54	17.30		
CINIII	292	104	35.62	12.20		
Cervical cancer	8	1	12.50	13.60		
Treatment mode					10.06	<0.01
Naught	75	24	32.00	23.20		
Pharmacotherapy	207	104	50.24	20.40		
Surgical treatment	326	109	33.44	13.10		
Number of multiple infections					2.563	0.11
Single infection	424	180	42.45	16.20		
Double infection	138	45	32.61	21.30		
Triple infection	38	11	28.95	26.70		
Quadruple and above	8	1	12.50	INF		
Type of infection					1.182	0.277
LR-HPV infection	27	11	40.74	23.90		
HR-HPV infection	529	206	38.94	16.30		
HR and LR HPV mixed infection	52	20	38.46	23.20		
Occupation					4.196	<0.05
Employees in the corporate, business, and service sectors	39	17	43.59	25.80		
Industrial workers	99	34	34.34	14.80		
Farmers	155	59	38.06	14.70		
Others	181	89	49.17	17.60		
Personnel in government agencies, party organizations, and public institutions	46	13	28.26	29.40		
Unemployed individuals	88	25	28.41	15.80		

#### 3.2.2 Cox multivariate analysis

A multivariate Cox proportional hazards model was constructed, incorporating factors identified as statistically significant in the univariate analysis. Multivariate Cox regression analysis revealed that age (HR = 1.71, 95% CI 1.28–2.28), infection status (single infection vs. multiple infection; HR = 0.68, 95% CI 0.49–0.93), and medical insurance type (HR = 0.74, 95% CI 0.56–0.98) were independently associated with the outcome. In contrast, ethnicity, occupation, menopausal status, degree of cervical lesions, and treatment approach did not demonstrate significant associations in this multivariate analysis. as shown in Table 4.

**Table 4:** 608 cases of HPV infection female outcome of COX multivariate analysis.

Variable	*HR*	95% CI	*p*
Age			<0.01
35–50		Reference	
<35	1.71	1.28 ~ 2.28	
>50	1.34	0.69 ~ 2.60	0.3914
Ethnic group	1.42	0.65 ~ 3.13	0.3786
Occupation	0.99	0.92 ~ 1.06	0.6898
Type of medical insurance	0.74	0.56 ~ 0.98	<0.05
Menopause	0.86	0.48 ~ 1.54	0.6145
Infection status (single/multiple)	0.68	0.49 ~ 0.93	<0.05
Degree of cervical lesion	1.09	0.94 ~ 1.26	0.2653
Treatment mode	1.25	0.93 ~ 1.69	0.1389

## 4 Discussion

Variations in HPV incidence, clearance, and persistence have been observed across different regions and countries (26–28). Domestic studies (3, 17) have shown that about 50% of HR-HPV-positive women with normal baseline cytology clear the infection through autoimmunity within 2–3 years without progressing to persistent infection.

This study’s follow-up data further highlight the complexity of HPV clearance. Among 608 HPV-infected women, the HPV clearance rate was 38.98%, with a median clearance time of 8.95 months, which is slightly longer than that reported in similar studies, potentially due to differences in study subject characteristics and follow-up methods. Previous studies have reported varying clearance rates and times. For example, Song et al. (29) reported a 68.9% clearance rate within 6 months among 173 cervical cancer patients in Beijing, China. Abudurexiti et al. (10) reported a median clearance time of 10.4 months among 97 individuals diagnosed with high-grade squamous intraepithelial lesions (HSIL) as well as 437 individuals with early-stage invasion (CC, Stage IA-IIA) in Xinjiang. Although the median clearance time of patients with HSIL was longer (*p* < 0.05), no significant differences were found between the HSIL grades, CC stages or age groups (*p* > 0.05). Bogani et al. (30), in a multi-center study of 3,212 women, reported that CIN3 patients had a significantly higher persistent HPV infection rate than CIN2 patients did (OR: 3.80, 95% CI 2.01–7.21). A systematic review and meta-analysis (31) revealed an increased risk of CIN1 progression due to persistent HR-HPV infection. However, Arthur et al. (32) reported no significant difference in the duration of HPV infection across different degrees of cervical lesions. Similarly, while the univariate analysis in this study revealed an association between cervical lesion degree and HPV clearance, the multivariate analysis did not confirm this association, potentially because of differences in population selection, study design, and detection methods. The relatively low HPV clearance rate observed in this study may be attributed to natural differences in clearance rates among study populations, low patient follow-up rates, and small sample sizes. Future studies should continue to follow this cohort and collect additional data to draw more comprehensive conclusions.

Previous research has consistently identified age as a crucial factor influencing both the acquisition and outcome of HPV infection. This study’s findings support this view, demonstrating that HPV-infected women aged younger than 35 years presented the highest rate of negative conversion (46.6%), whereas those over 50 years of age presented the lowest rate (28.41%). Similar findings have been reported by Li et al. (33), who revealed that advanced age was associated with a greater likelihood of persistent HPV infection (OR = 2.73, 95% CI = 1.07–6.93) in a one-year follow-up study of 152 baseline HPV-positive women in Daqing city. Feng et al. (34) research also revealed that age is an independent variable that influences the natural resolution of high-risk HPV (HR-HPV) infections among women with normal or low-grade cytology, with clearance rates generally being lower in older individuals. However, conflicting results have also been reported. Pan et al. (35) found that age was an independent risk factor for persistent HPV infection; however, paradoxically, HPV conversion rates were lower in women over 45 years of age than in other age groups. These discrepancies may be attributed to variations in study design, follow-up duration, age distribution, sample size, individual immune levels, hormone levels, and other factors.

Our study included 17 variables, of which 8 factors showed statistical significance in univariate analysis. In the multivariate analysis, three variables (age, medical insurance type, and infection status) were found to be associated with HPV clearance, while five variables (ethnicity, occupation, menopausal status, cervical lesion severity, and treatment method) did not show a significant relationship with HPV negativity. However, previous studies have indicated that factors such as ethnicity, occupation, menopausal status, cervical lesion severity, and treatment methods may influence HPV clearance outcomes. It is worth noting that this study was conducted in an area predominantly inhabited by Han people. In contrast, ethnic differences may be more pronounced in regions with a larger presence of minority populations. The lack of a significant association between occupation and HPV clearance may be attributed to the diverse occupations of patients in the surrounding area. In Shenzhen, a region characterized by low aging, high education levels, and strong treatment adherence among postmenopausal women, these factors could contribute to the limited differences observed in our study. Additionally, most of the patients in this study were of adult and pre-menopausal age, which may explain the lack of variation in menopausal status, cervical lesion severity, and treatment outcomes. Nevertheless, differences in regional characteristics and research methodologies may explain some of the discrepancies between our findings and those of other studies.

The age distribution of patients with HPV infection is bimodal, as demonstrated by several studies. A multi-center cross-sectional study in China (36) revealed that HR-HPV infection rates exhibit two peaks, during adolescence and the perimenopausal period, with the highest prevalence observed among women aged 15–24 and 40–45. The secondary peak may be attributed to increased HPV exposure resulting from aging and alterations in the internal environment and immune system. Furthermore, HPV infection rates in urban and rural areas display distinct trends with age (37, 38). A three-year follow-up study on HR-HPV infection and the risk of cervical lesions in women with normal baseline cytology revealed that the HR-HPV negative conversion rate was consistently higher in women aged younger than 45 years than in those aged older than 45 years (17). These findings indicate that age significantly influences the natural history of HPV infection. This pattern potentially correlates with the decline in immune function attributed to aging, which may decrease the efficacy of the body in eliminating recent HPV infections or suppressing latent HPV. However, the relationship between age and HPV conversion requires confirmation in larger samples. Given that middle-aged and older adult women are more likely to experience persistent HPV infection, strengthening clinical follow-up and encouraging regular return visits are recommended to increase monitoring and intervention effectiveness.

Currently, the application of more perfect cervical cancer screening strategies and the results of HPV tests with higher analytical sensitivity in cervical cancer screening have focused on the detection of multiple HPV infections. Studies have shown that the incidence of HPV multiple infections is higher than expected, accounting for about 20–50% of HPV-positive patients (39). At present, research on multiple HPV infections has focused on two main aspects: the interaction of different types of HPV in multiple infections and its influence on persistent infection, and the impact of multiple infections on the risk of future cervical diseases. The results of this study revealed that HPV infection was dominated by a single infection (71.22%), whereas Chen et al. (40) reported that the HPV infection rates and subtype distributions of 326,824 cervical cancer screening and health examination subjects in Hunan Province were essentially the same, with a single infection accounting for 73.18% of the total. Furthermore, the research indicated that women with a solitary infection presented a negative conversion rate of 43.19%, markedly surpassing the 28.57% reported in those with multiple infections. However, the study failed to find a significant association between multiple infections and the risk of cervical lesions.

Multiple studies have shown that, compared with a single HPV infection, multiple HPV infections significantly increase the risk of cervical lesions. A study in South Korea (41) revealed that women with multiple HPV infections were more likely to develop persistent HPV infection than women with a single HPV infection and were more strongly associated with high-grade cervical lesions. Brot et al. (42) reported that multiple HR-HPV infections are associated with ongoing low-grade squamous intraepithelial disease (LSIL). In contrast, Bruno et al.’s research (43) indicated that the risk of single HPV infection with cervical squamous cell carcinoma (SCC) is greater than that of multiple infections, which are associated mainly with early cervical lesions (CIN1–CIN2). Luo et al. (44) reported that the associations between multiple HPV infections and high-grade squamous intraepithelial disease (HSIL) and LSIL were stronger in 20,059 women undergoing physical examination in Chongqing, China, and certain types of combinations may synergistically increase the risk of HSIL and LSIL. The findings of these studies may be attributed to the independent pathogenicity theory of HPV (15, 45), where multiple infections accumulate individual risks without interactions between HPV types. Conversely, synergies between HPV subtypes could link multiple infections to persistent infections, leading to more severe cervical lesions.

Although some studies suggest that multiple infections increase the risk of cervical lesions, others find no significant effect. Zhong et al. (46) analysis of 7,036 cases from 2018 to 2022 revealed no association between multiple HR-HPV infections and an increased risk of CIN2 development. Another study showed (47) that the combination of HPV16 with other genotypes reduced its pathogenicity, potentially because of competitive interference rather than synergistic or cumulative carcinogenesis at the phylogenetic level. Multiple infections may stimulate a rapid immune response, shortening the HPV clearance time and improving clearance rates (48, 49).

A Chinese study suggests potential interactions between HPV vaccine types and non-vaccine HPV types, with possible type replacement occurring following vaccination (15). Some studies propose that antagonistic interactions may exist between different HPV genotypes in cases of multiple infections (39, 45, 50). Conversely, other research indicates that multiple infections might exhibit synergistic effects. For instance, certain combinations of HPV genotypes accelerate the virus’s transcription, while others inhibit it. As a result, the relationships between HPV types in multiple infections are complex and variable. We hypothesize that the genotypes present in multiple infections may involve: (1) a persistent genotype, (2) a new genotype that initially antagonizes the pre-existing one, or (3) a cross-infection between the previous and newly acquired genotypes (51).

In this research, the infection rates of HPV16 and HPV52 were the highest, at 27.632 and 27.467%, respectively, making them the most prevalent types. In terms of clearance rates, HPV6, HPV40, and HPV54 exhibited relatively high negative conversion rates of 62.5, 66.7, and 66.7%, respectively, indicating favorable clearance outcomes. In contrast, HPV35 had the lowest negative conversion rate, at only 18.75%, suggesting that the likelihood of clearance for this type is relatively low. Studies (52) have revealed that the clearance times of different HPV types vary, with high-risk (HR) HPV types, such as HPV16 and HPV18, generally having longer clearance times than low-risk (LR) types. This extended clearance time makes HR-HPVs more dangerous. In cases of multiple infections, the combination of different HPV types results in varying clearance times among individuals, compared to single infections. Currently, both preventive and therapeutic HPV vaccines target specific HPV types. While some HPV types may be cleared more quickly in individuals with multiple infections, other types remain active, potentially promoting further infections. Therefore, more research is needed to understand the dynamics of multiple infection types and their relationship with HPV clearance time. Future studies should explore the activation and elimination mechanisms of different genotype combinations, the effects of various treatments on HPV clearance, and the identification of variant lineages through genetic sequencing.

China’s rapid economic development and urbanization have prompted a surge in cross-city population mobility, particularly between major cities such as Beijing, Shanghai, Guangzhou, and Shenzhen and neighboring provinces (53–55). The hukou system, implemented in the 1950s, restricts geographical mobility and defines access to employment, housing, social welfare, and education. Internal migrants (IMs), individuals relocating from their domicile cities, often face difficulties using medical insurance across regions because of differences in coverage between their home cities and current residences (54, 56, 57). Although the government has issued relevant policies to simplify the process of using cross-regional medical insurance, IMs still face some problems in practical use, such as difficult reimbursement, cumbersome procedures and insufficient coverage of community medical services (58, 59). These restrictions often cause IMs to rely on cross-provincial medical insurance settlements or self-funded medical treatment, which increases the economic burden and further affects medical decision-making.

The multivariable analysis conducted in this study revealed a substantial correlation between the type of health insurance and HPV infection outcomes. A cross-sectional study conducted by Holt et al. (55) among women who were either migrants or non-migrants aged 21–65 from seven Chinese provinces revealed that employer-provided medical insurance enhances cervical cancer screening uptake among the migrant female population in China. Furthermore, the scope and type of medical insurance significantly influence diagnostic and treatment investments, as well as ultimate medical decisions (60). Research by Zhao et al. (61) in 10 districts in Shenzhen revealed that patients with low-level medical insurance tend to opt for community health service centers for graded diagnosis and treatment. A study at a community health service center in Shenzhen’s Bao’an (62) district revealed that medically insured women were more likely to register with family doctors and actively participate in community-based cervical cancer screening and follow-up programs. These findings align with international research on migrant workers (63). Improving the medical insurance system, particularly for migrant workers, is crucial for enhancing their disease prevention and diagnosis participation. Such reforms are expected to alleviate the national economic burden while increasing the negative conversion rate of HPV infection and improving overall migrant population health.

Access to healthcare across different regions is influenced by a range of factors, including health, social, and economic conditions. Consequently, the conclusions drawn in this study regarding the relationship between medical insurance and HPV clearance time should be viewed with caution, as our research does not establish a clear causal link between the two. While the reasons for population movement differ between European countries and cities within China, migrants face similar challenges in accessing healthcare: disparities in access to primary care at various levels of the healthcare system. Even in countries with well-established healthcare systems, immigrants often encounter significant barriers to obtaining health services. Studies from Italy, Norway, and Denmark (64–66) have shown that immigrant participation in cervical screening is lower than that of local populations. However, some research in China (55) suggests that, unlike local residents, the participation of migrant women in cervical cancer screening is not directly linked to their medical insurance status. These women’s families often purchase private insurance for them, allowing them to afford preventive screenings and treatment. It is therefore speculated that the economic situation of migrant families, along with mutual support among family members, may mitigate the impact of limited access to medical insurance, potentially enhancing HPV clearance rates.

There is a notable gap in preventive healthcare access between immigrants and local populations. To encourage greater participation in disease management, it is crucial for health authorities to implement policies that reduce barriers to HPV treatment among migrant populations. Healthcare services should develop more tailored and inclusive programs, particularly for rural migrants living temporarily in cities. Long-term, evidence-based prevention programs that are regularly evaluated are essential for migrants. Furthermore, leveraging Shenzhen’s institutional strengths, with hospitals serving as central hubs and community health services as a foundation, public health policymakers could prioritize HPV screening and vaccination programs for the most vulnerable groups in the area. This would help alleviate the healthcare burden on both hospitals and patients (67, 68). It is also recommended that government agencies and healthcare institutions intensify public awareness, education, and training for both the migrant population and healthcare providers, focusing on HPV treatment and cervical cancer prevention.

Studies have also shown (69) that the prevalence of HPV infection increases in women over the age of 50, as immune system effectiveness declines with age. This decline in immunity makes older women more vulnerable to persistent HPV infections or reactivation of latent viruses. HPV vaccination is believed to play a positive role in promoting HPV clearance. While our study did not collect data on HPV vaccination status in women, research from various countries indicates that preventive vaccination is effective in facilitating HPV clearance. One study demonstrated that HPV vaccination significantly reduced persistent HPV infections and associated cervical intraepithelial neoplasia (CIN) in women aged 18 to 25 years (70). Other studies have also highlighted the benefits of vaccination for women aged 40–45 years and beyond, suggesting that vaccination can still protect against HPV infections and related conditions, such as cervical cancer, in older women. Although our study did not include young girls (14–18 years old), several studies suggest that completing the HPV vaccination program before puberty offers significant benefits. Vaccination is particularly important for young women to undergo before their first sexual activity (< 30 years of age). Furthermore, women over 50 should be regularly screened for cervical cancer (71–73).

Although our study does not explore lifestyle factors, existing research suggests that lifestyle may influence HPV clearance. Specifically, similar lifestyle patterns before and after HPV infection may provide common transmission routes and risk factors for persistent or cross infections. HPV is primarily transmitted through sexual contact. Due to traditional views, the number of men who proactively undergo HPV screening remains low, and those without symptoms are often reluctant to seek treatment. As a result, even if women are treated for HPV, continued sexual activity with untreated male partners increases the risk of persistent infection. Furthermore, a study on the quality of life for women with pre-and postmenopausal HPV infections (74) found that postmenopausal women with HPV reported a lower quality of life, particularly those experiencing sexual disorders. These changes were linked to mental health issues, with the dual pressures of recovery and marital sexual demands contributing to psychological strain, which may hinder the process of HPV clearance.

With respect to contraceptive methods, condom use was reported by less than 20% of the 608 patients; thus, these patients were excluded from the analysis. The relationship between intrauterine device (IUD) use and persistent HPV infection remains debated (75). This study revealed a significantly lower HPV conversion rate among IUD users than among nonusers. However, multifactorial analysis revealed no significant correlation, precluding a definitive conclusion about the impact of the IUD on HPV infection outcomes. Further investigation is warranted to elucidate the potential link between the use of IUDs and the clearance of HPV infections.

## 5 Summary

This retrospective study analyzed 608 women with HPV infection from Shenzhen Longgang Central Hospital. The results revealed that a greater proportion of women aged 35–50 years at initial diagnosis had a single HPV infection and high-risk HPV infection. Multiple factor analyses revealed that age, type of medical insurance, and type of infection were independent factors influencing the conversion rates of HPV-negative patients. These findings underscore the importance of early screening, detection, and diagnosis of cervical cancer at the grassroots level. Increasing public awareness of cervical cancer screening, issuing timely health risk warnings to high-risk female groups, and ensuring thorough follow-up of HPV-infected patients are crucial measures. The coordinated development of medical and preventive measures is essential to mitigate the burden of HPV-associated cervical diseases in China. Effective implementation of these strategies will be critical in reducing the disease burden in the future.

This study is still inadequate. Owing to the limitations of a single-center retrospective study, the results can only represent the outcomes of HPV-infected women in Shenzhen but can provide new insights for the follow-up of HPV prognosis in other first-tier cities. Data on sexual behavior and HPV vaccination status were not gathered in this research, thereby limiting the ability to assess the impact of these significant factors on HPV outcomes. The degree of cervical lesions considered among the 17 variables in this study was time dependent, but the effect of baseline conditions on HPV infection outcome was analyzed only in this study, which may have skewed the results. In addition, there have been previous studies on menopausal status, lesion degree, and treatment. Although the single-factor analysis of this study revealed correlations, the multifactor analysis results revealed that multiple factors were not independent influencing factors, which was considered to be related to the uneven distribution of the population with relevant characteristics included in this study. In the future, high-quality, large-sample prospective studies should be carried out, the quality of life and anxiety and depression scale scores should be increased, and the diagnosis and follow-up plans for HPV-infected patients should be improved for clinical reference.

## Data Availability

The raw data supporting the conclusions of this article will be made available by the authors, without undue reservation.
